# The Significance of Artificial Intelligence Platforms in Anatomy Education: An Experience With ChatGPT and Google Bard

**DOI:** 10.7759/cureus.45301

**Published:** 2023-09-15

**Authors:** Hasan B Ilgaz, Zehra Çelik

**Affiliations:** 1 Anatomy, Hacettepe University Faculty of Medicine, Ankara, TUR

**Keywords:** chatgpt, large language models, google bard, artificial intelligence, anatomy education

## Abstract

This study evaluated the use of two large language models (LLMs), ChatGPT and Google Bard, in anatomy education. The models were asked to answer questions, generate multiple-choice questions, and write articles on anatomy topics. The results showed that the models were able to perform these tasks with varying degrees of accuracy. ChatGPT and Google Bard did not differ significantly in terms of answering questions. Both models were able to generate multiple-choice questions with a high degree of accuracy. However, the performance of the models in article writing was not yet at a sufficient level. The study also found that the use of LLMs in medical education requires caution. This is because LLMs are still under development and they can sometimes generate inaccurate or misleading information. It is important to carefully evaluate the output of LLMs before using them in educational settings. Overall, the study found that LLMs have the potential to be valuable tools for anatomy education. However, more research is needed to improve the accuracy of the models and to better understand how they can be used effectively in educational settings.

## Introduction

The development of large language models (LLMs) is a major advance in artificial intelligence (AI) and natural language processing (NLP). LLMs have the capacity to process and understand natural language data with extraordinary skill and are already used in a variety of applications such as chatbots, machine translation, and question-answering [1-3]. Some of the most popular LLMs are ChatGPT and Google Bard, both of which have garnered a lot of attention for their capacity to understand textual information and generate contextually relevant responses [4]. These applications are being tried to be used in medical education. Recently, numerous articles have emerged about AI applications in different fields of medicine such as radiology, dermatology, physiology, hematology, ophthalmology, biochemistry, parasitology, neurosurgery, forensic medicine, dental education, etc. [5-14]. These models have been helpful in solving complex medical problems, interpreting radiology reports, being used to diagnose diseases, writing scientific articles or answering and generating different medical exam questions and have shown varying degrees of accuracy in these fields [2-5,7,8,10,11,15]. Although there are not enough articles about its use in the field of anatomy, there are some preliminary evaluations [16-21]. The present study aims to evaluate how various features of open AI platforms such as ChatGPT and Google Bard in their current form can contribute to anatomy education with the perspective of questioning, answering, and writing articles, and aims to respond to some controversial topics.

## Materials and methods

Data collection

In order to evaluate the usability of the applications in anatomy education, three stages were first defined. In the first stage, the answers of the applications to the anatomy exam questions would be evaluated. In the second stage, their ability to form questions and in the last stage to write scientific articles would be evaluated. For these processes to be applied sequentially, user accounts were first created for both ChatGPT (https://chat.openai.com/) and Google Bard (https://bard.google.com/) chat boards. Then for the first stage, ChatGPT and Google Bard platforms were requested to answer the last five years of the Medical Specialty Exam (MSE) questions. MSE is the main specialization exam for medicine in Türkiye and is held twice a year by The Measuring, Selection and Placement Center (OSYM - A Governmental Organization). The anatomy sections of the MSE were about 14 questions (2021-2 MSE questions were given as an example in *Appendix 1*). As it could not be reached, the first exam in 2023 and the two exams in 2022 could not be evaluated. In order to evaluate whether the language option affects the success of the chat boards, the questions were also translated into English through them. After the translation process was done by the chat boards, it was checked by two anatomists (one of them is an anatomy specialist and the other one is an anatomy specialist student). The questions that were found to be wrong or insufficient were corrected by the evaluators and asked on the chat boards. Two questions in ChatGPT and one question in Google Bard were corrected due to syntax and grammar errors. Since the software cannot evaluate visual content, all questions except those with visual content were asked. A total of 131 questions were asked on the chat boards for each user. The users were ChatGPT Turkish 1, ChatGPT Turkish 2, ChatGPT English, Google Bard Turkish 1, Google Bard Turkish 2, and Google Bard English. The accuracy of the answers was assessed according to the existing literature. The correct answers to Turkish and English questions were compared. A second user was created in both apps to assess whether the responses to the questions were systematic or whether the apps taught random answers. When these answers were compared, it was also observed whether asking the same questions resulted in more accurate answers in the second answer, thanks to machine learning. The accuracy rate of the answers given by the first and second users created in the same chat board was evaluated by comparing them with each other. In the next step, the question-answer capabilities of the software were evaluated. ChatGPT and Google Bard were asked to create and answer MSE-level multiple-choice anatomy questions. Almost all of them were correctly constructed. We asked about 100 questions for 40 different topics and only four of them were wrong. These 40 topics were identified by two anatomists. In order not to expand the boundaries of the article too much, instead of 40 topics, three random topics were sampled in the article. Then, the level was adjusted by giving the commands "make the question harder" or "recreate this question" for those who did not have the appropriate content. The MSE level emerged with the experience of two anatomists studying the exam questions of the last 10 years. The accuracy of the questions and answers at all stages was evaluated according to the current literature. And, finally, the software was asked to describe its ability to write academic papers. The validity of the anatomical information in the paper was checked, and the evaluation of whether it was detailed enough as a scientific article was done by the two different anatomists.

The free version of the LLMs’ was used (ChatGPT 3.5 and Google Bard). All the data were collected from ChatGPT and Google Bard.

Statistical analysis

All data were analyzed by using the IBM SPSS (Statistical Package for Social Sciences - Armonk, NY: IBM Corp) version 22.0 package. The data were expressed as numbers and percentages. For the analysis of results, two simple t-tests were used for parametric variables, while the Mann-Whitney U test was used for non-parametric variables. Kruskal Wallis test was used to evaluate the differences between all groups. The significance level was set at p < 0.05.

## Results

Answering questions

**Figure 1:**
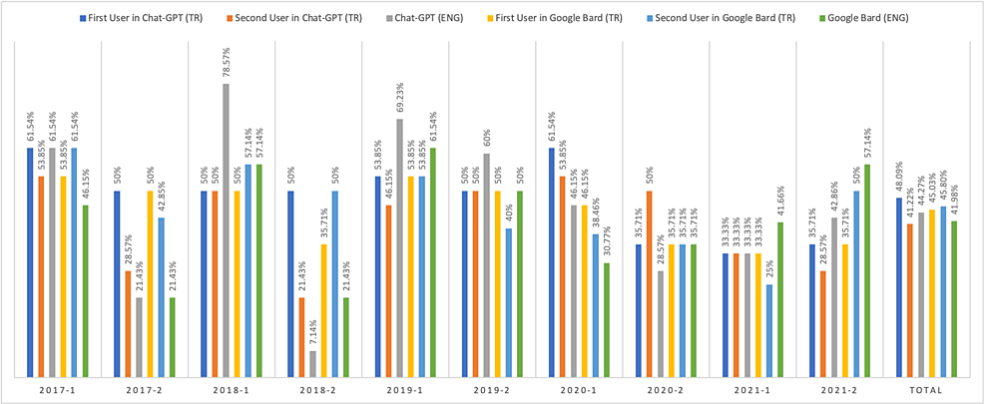
Results of the answering questions. (TR: Turkish, ENG: English)

The scores obtained after asking questions to the applications are shown in Figure 1. The most successful results were seen in the first user of ChatGPT in Turkish (48.09%), the second one was the second user of Google Bard in Turkish (45.80%), and the others were first user in Google Bard in Turkish (45.03%), ChatGPT in English (44.27%), Google Bard in English (41.98%) and first user of ChatGPT in Turkish (41.22%), respectively. None of them scored above 50% on the total correct answers. When the overall results were compared in all groups, no statistically significant difference was found (p>0.05). 

Question generating

**Figure 2:**
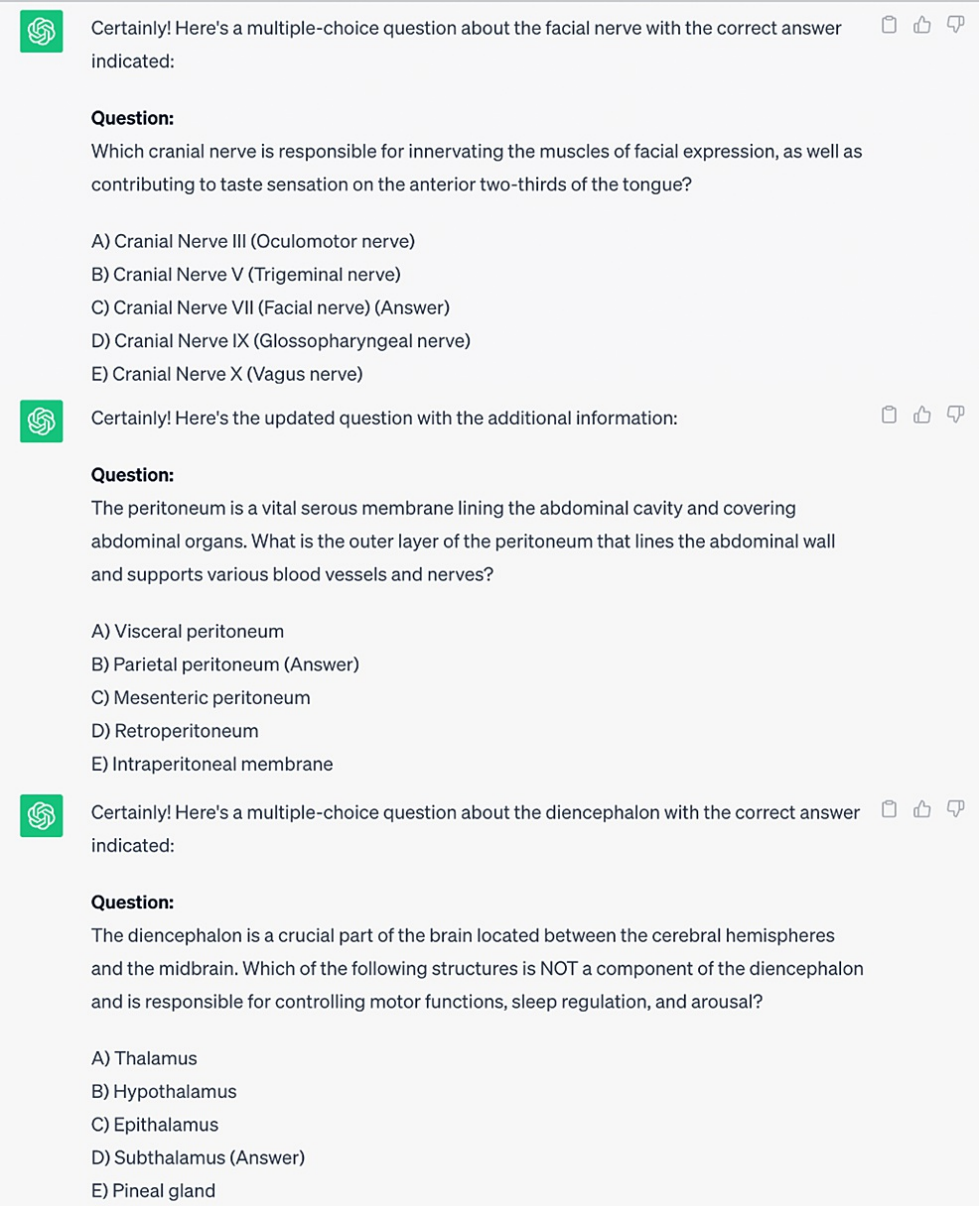
Questions and answers created by the ChatGPT.

At this stage, the software was tasked with preparing three sample questions about facial nerve, peritoneum, and diencephalon anatomy which were selected by the two anatomists. The questions and answers created by the ChatGPT are shown in Figure 2.

The answer to the third question generated by ChatGPT was wrong [22].

**Figure 3:**
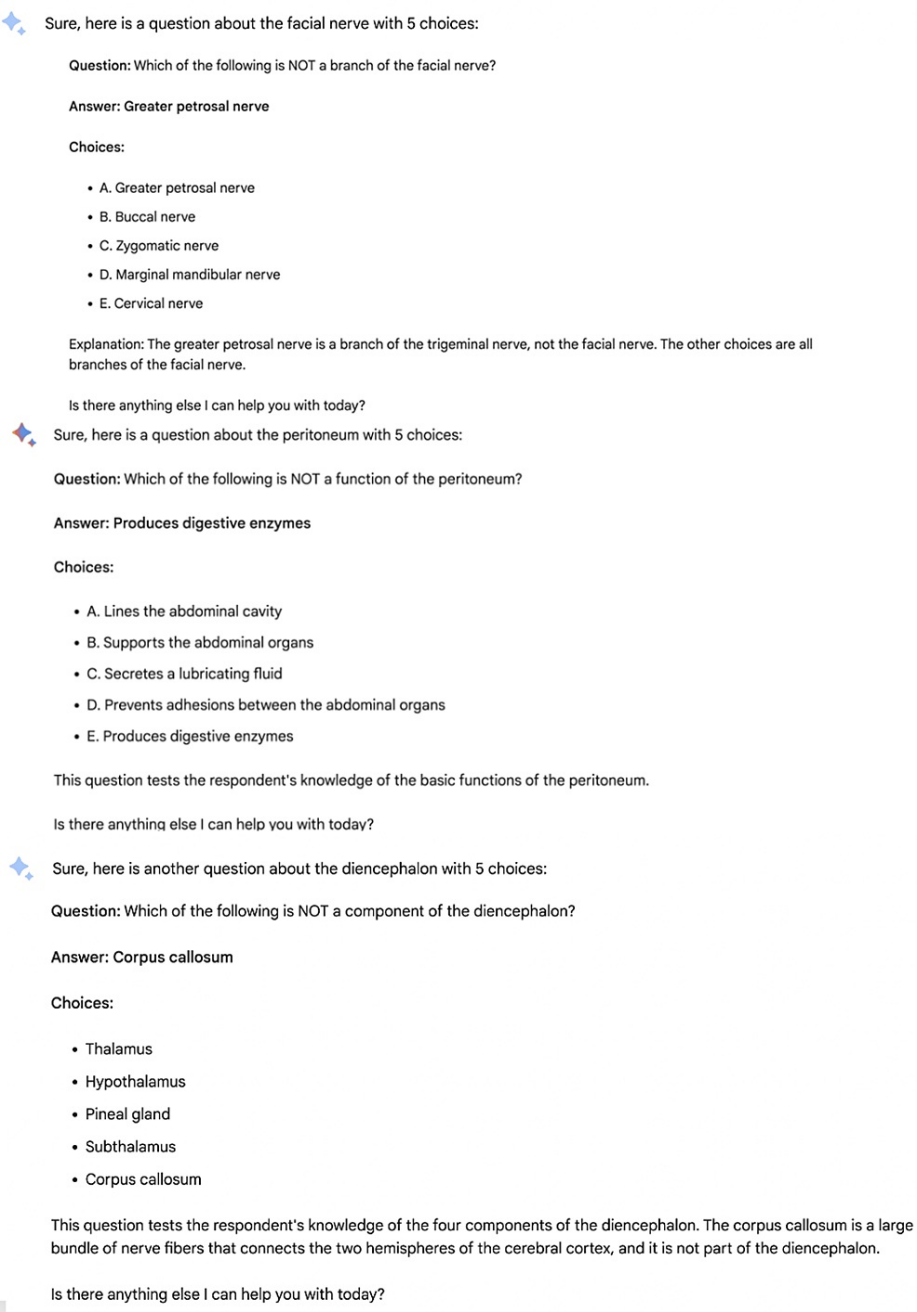
Questions and answers created by the Google Bard.

The questions and answers created by the Google Bard are shown in Figure 3.

All the answers to the questions generated by ChatGPT were correct [22].

Article writing

**Figure 4:**
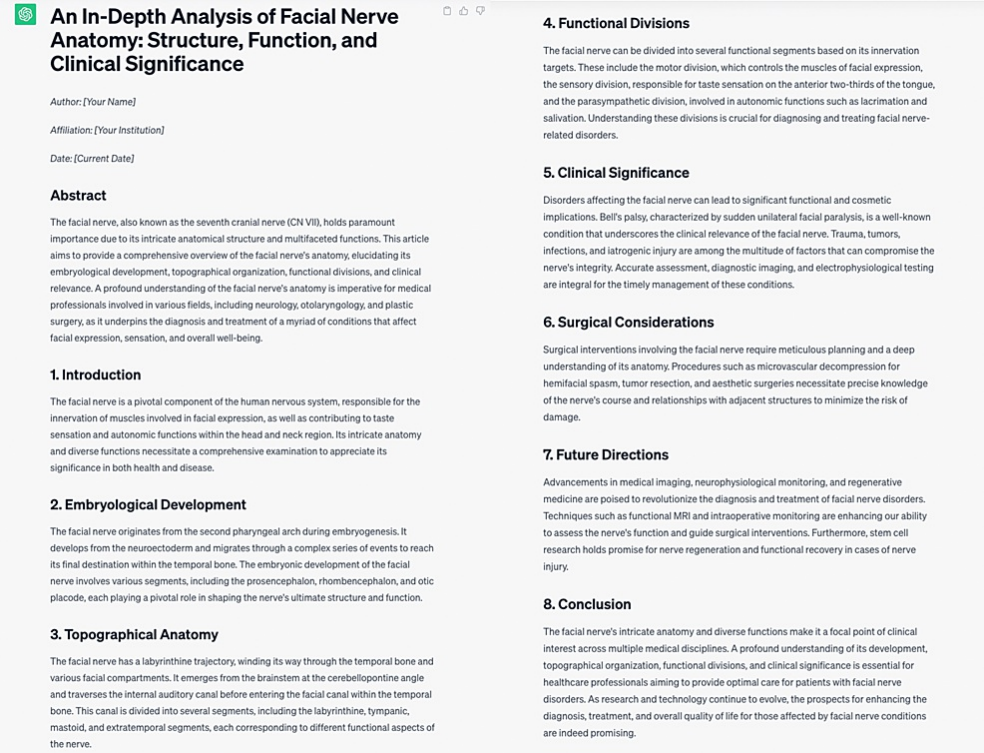
The article written by ChatGPT.

The models were asked to write an academic paper on the anatomy of the facial nerve. The article written by ChatGPT is shown in Figure 4.

**Figure 5:**
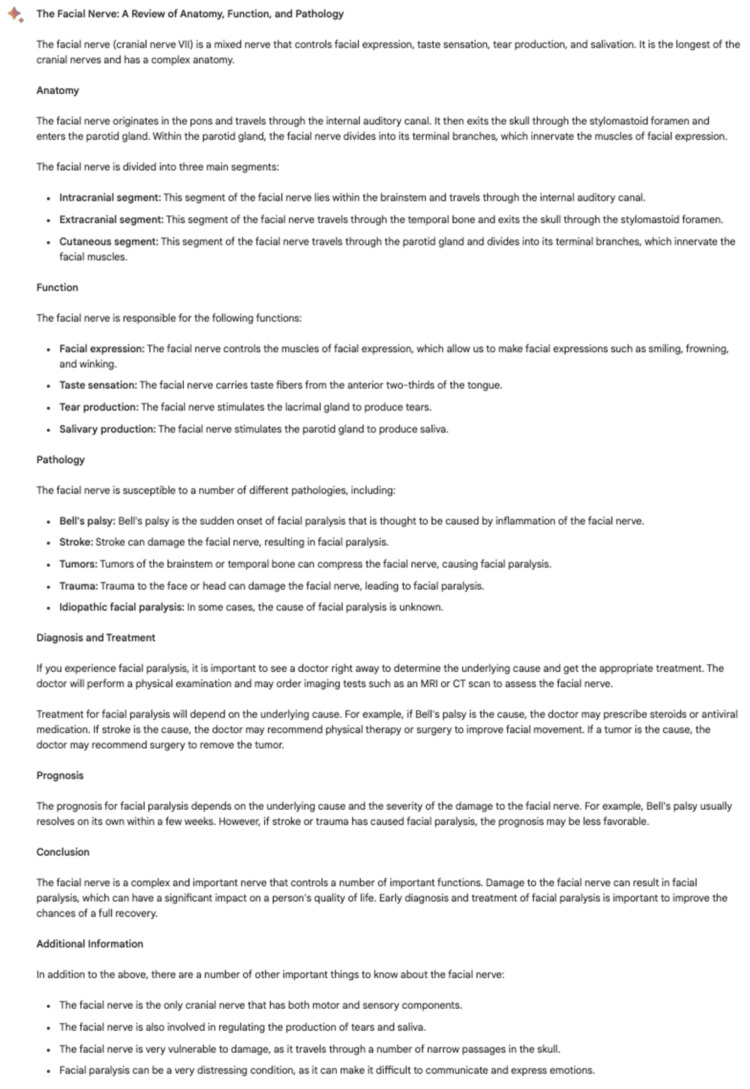
The article written by Google Bard.

And, the article written by Google Bard is shown in Figure 5.

## Discussion

Although artificial intelligence applications are widely used in the field of medicine, there is not enough evaluation in the field of anatomy and anatomy education yet. Mogali used ChatGPT in the fields of asking questions, engaging like a tutor, and writing assessment items, and as a result, states that although his contribution to students and teachers in type education is not yet at a sufficient level, it has a potential for development [16]. Similar to Mogali’s assessment, ChatGPT has significant potential to enhance student's learning experiences and create a more interactive and engaging educational environment, according to Lee, in an article that looked at it by responding to directions and figuring out the advantages and disadvantages of it and designing a curriculum [18]. Totlis et al. on the other hand, by asking 18 questions about anatomy in the fourth version of ChatGPT, stated that this model can be a valuable interactive educational tool that encourages interaction, but that it cannot replace the critical role of educators and should be used as a complementary tool [17]. Abdellatif et al. and Lazarus et al., similar to the evaluations in other studies, also stated that these LLMs have great potential in anatomy education, even if they are not yet at a big efficiency [19,20]. Li et al. customized a database to train an artificial intelligence dialogue system for teaching anatomy to medical students in real-time by using an open-source machine learning architecture, they conducted a preliminary study. After students' practice sessions, they reported an increase in students' confidence in anatomy knowledge from 2.10 to 3.84 on a five-point Likert scale [21]. 

Similar to these studies, we observed that ChatGPT and Google Bard, which we evaluated in terms of generating questions, responding to questions, and writing scientific articles in our study, have a significant potential for improvement in anatomy education for medical students and medical teachers in the future.

In addition, when we compared ChatGPT and Google Bard models, we found that there was no difference in problem-solving skills in anatomy. Both chat boards correctly answered about half of the anatomy questions asked. We used ChatGPT-3.5 in our study. Moshirfar et al., on the other hand, used the fourth version of the ChatGPT in their study and found that the fourth version was more successful in answering the questions [9]. The success of the fourth version was both a limitation of our work and proof that chat boards could be developed in a better way. Another limitation of this study was that we could not make visual evaluations, which are crucial in anatomy education. There is a need for future studies on the application of 2D and 3D figures in anatomy education with other artificial intelligence applications. 

LLMs are evolving every day. Therefore, more studies are needed to fully explore its capabilities in the future [16-20]. However, these abilities will develop through deep learning. When we tested the performance of the models in different languages, we did not observe any difference between ChatGPT and Google Bard here either. We evaluated that the dataset of these data software did not make any difference in response to the questions in different languages, which showed that the language options were working well. We also tested that the success does not increase if the questions are repeated as a result of machine learning, by creating two different users. Furthermore, we concluded that the algorithm of the software is open to learning from the same user and that no progress was made when the same questions were asked by different users at different times. These results highlight the varying proficiency of language models in reaching accuracy with the same questions at the same time. 

When we evaluated the generating questions, we found that both ChatGPT and Google Bard were quite good. Even though ChatGPT generated one incorrect question, these two chat boards can be used to make exam questions of different difficulty and can save teachers time. In fact, Google Bard can be considered a little better in this regard, because while preparing a question, it also suggests different alternative questions. 

In terms of creating a scientific article, we saw that both chat boards article writing ability was less detailed, without references, and at a basic level, compared to previously written scientific articles on the subject. ChatGPT has created an article that is more in line with academic norms on this subject, with its general article structure and information divided into subheadings such as embryological development, clinical importance, surgical issues, future directions, and conclusion sections.

The model has the potential to become a more reliable and valuable tool for anatomy education and exam preparation by improving the ability to understand anatomy-related concepts and generate appropriate questions and distractors for different difficulties.

## Conclusions

There were differences in LLM performance, but no significant difference was observed between ChatGPT and Google Bard in terms of answering questions. The question generating of these models was satisfied. It can be useful for exam preparation in educational aspects. The performance of the applications in article writing was not yet at a sufficient level. While LLMs show promise as valuable tools for medical education, their use requires caution, given the complex algorithms and their limitations in dealing with potential inaccuracies. This study highlights the need for continuous improvement and validation of LLMs for reliable healthcare practices. Further research is needed to increase the accuracy of the models and to better understand how they can be used effectively in educational settings.

## Appendices

Appendix 1

2021-2 MSE Questions

1. The physical examination of the patient, who applied to the emergency clinic with complaints of pain and swelling in his foot after falling from a tree, revealed widespread pain on the sole of the foot and collapse of the medial arch of the foot. The patient's lateral ankle radiography shows that the talar head is dislocated inferiorly.

Which of the following anatomical structures is most likely to be injured in this patient?

A) Plantar aponeurosis

B) Plantar calcaneonavicular ligament

C) Medial talocalcaneal ligament

D) Calcaneocuboid ligament

E) Long plantar ligament

2. Which of the following joints is considered within the plane type articular group, according to the shape and movements of the joint surface?

A) Acromioclavicular joint

B) Proximal radioulnar joint

C) Median atlantoaxial joint

D) Carpometacarpal joint of the thumb

E) Metacarpophalangeal joints

3. In a 68-year-old female patient who underwent a brachial plexus block developed winged scapula after the procedure. On physical examination, the scapula is observed to be elevated and slight medially rotated at rest.

Which of the following nerves is most likely to be injured during the brachial plexus block in this patient?

A) Accessory nerve

B) Dorsal scapular nerve

C) Suprascapular nerve

D) Long thoracic nerve

E) Axillary nerve

4. The inferior angle of the scapula is commonly used as a superficial anatomical landmark in physical examination.

In an adult which stands on the standart anatomical position, at which vertebral level is the angulus inferior located?

A) T3

B) T5

C) T7

D) T9

E) T11

5. Which of the following regions is the most appropriate for palpating the pulse of the posterior tibial artery?

A) Between the lateral and medial malleoli on the anterior side

B) Anterior to the lateral malleolus

C) Posterior to the lateral malleolus

D) Anterior to the medial malleolus

E) Posterior to the medial malleolus

6. Tentorial herniation is detected in a 35-year-old female patient who applied to the hospital with inability to move her right eye up, down and medially.

Which of the following nerves is most likely affected by the compression causing the clinical presentation in this patient?

A) Optic nerve

B) Oculomotor nerve

C) Trochlear nerve

D) Trigeminal nerve

E) Abducens nerve

7. For a minor surgical intervention on the lateral side of the foot, a surgeon injects subcutaneous local anesthetic just behind the lateral malleolus. Which of the following nerves is the most likely target of this block?

A) Posterior tibial nerve

B) Deep fibular nerve

C) Saphenous nerve

D) Superficial fibular nerve

E) Sural nerve

8. Which of the following pathways carries conscious proprioceptive, two-point discrimination, and vibration senses of the body?

A) Anterior spinothalamic tract

B) Gracile and cuneate fasciculi

C) Spinotectal tract

D) Posterior spinocerebellar tract

E) Posterior spinothalamic tract

9. Which of the following sinuses is the dura mater venous sinus, which plays a role in the circulation of the cerebrospinal fluid and has the most “granulationes arachnoideae”?

A) Superior sagittal sinus

B) Inferior sagittal sinus

C) Sigmoid sinus

D) Inferior petrosal sinus

E) Cavernous sinus

10. Which of the following is the free inferior border of the quadrangular membrane?

A) Vocal ligament

B) Vestibular ligament

C) Aryepiglottic fold

D) Thyroepiglottic ligament

E) Elastic cone

11. Where is the most common site of fertilization of the ovum by the spermatozoon?

A) Infundibulum of the uterine tube

B) Fimbriae of the uterine tube

C) Ampulla of the uterine tube

D) Isthmus of the uterine tube

E) Abdominal ostium of the uterine tube

12. Which anatomical structure, entering the inguinal canal from the deep inguinal ring and extending to the subcutaneous tissues of the labia majora pudendi, is a guide region for a minor surgical procedure on the lateral side of the foot?

A) Sacrotuberous ligament

B) Cardinal ligament

C) Rectouterine ligament

D) Pubocervical ligament

E) Round ligament of the uterus

13. During cholecystectomy, when the cystic artery is ligated, which of the following anatomical structures forms the medial border of the Calot's triangle?

A) Common bile duct

B) Cystic duct

C) Common hepatic duct

D) Left hepatic duct

E) Pancreatic duct

14. Which organ and its part are both sites of attachment for the lesser omentum and greater omentum?

A) Liver - Quadrate lobe

B) Stomach - Greater curvature

C) Stomach - Angular notch

D) Duodenum - Superior part

E) Duodenum - Inferior part
